# Supplementary material for: A Likelihood Approach for Real-Time Calibration of Stochastic Compartmental Epidemic Models
Source: PLoS Comput Biol. 2017 Jan 17;13(1):e1005257. doi: 10.1371/journal.pcbi.1005257 (PMC5240920; doi:10.1371/journal.pcbi.1005257)
Supplement: S1 File — (TAR.GZ) [file pcbi.1005257.s014.tar.gz › HSPH_Online-SI-Revision/EnKF07/n1-mild/n1-mild_EnKF_table0.pdf]

1	R0	{1.27677, 7.89375}	{2.32533, 4.8314}	{{0.895281, 0.212421}, {3., 11.0454}}	{98.6038, 59.7793}	inf	37.	53.2955	{7.46089, 107.665}	117.675	0.365319	newly	32.	68.42	{6., 236.}	176.063	0.424214	Reff	0.99205	0.457339	{0.119558, 0.908774}	59.4877	ar	400.	1769.98	{1620., 2150.}	342.495
2	R0	{1.33231, 10.5259}	{2.58455, 3.90625}	{{0.940059, 1.05181}, {3., 12.1438}}	{111.276, 70.7858}	inf	32.	83.13	{4.14478, 141.215}	216.577	0.456204	newly	17.	86.82	{5., 177.}	441.529	0.529767	Reff	0.766078	0.411492	{0.100195, 0.652349}	54.9607	ar	467.	1718.22	{1546., 1909.}	267.927
3	R0	{1.2273, 6.76944}	{2.5283, 5.32747}	{{2.13697, 1.60512}, {3., 9.65581}}	{108.879, 48.912}	inf	26.	91.3597	{21.6227, 204.163}	262.599	0.421872	newly	25.	137.5	{16., 319.}	464.64	0.517707	Reff	0.996568	0.683902	{0.0744863, 2.09679}	63.5656	ar	314.	1876.76	{1628., 2585.}	497.898
4	R0	{1.41215, 17.5742}	{2.80711, 8.36673}	{{2.44286, 6.15144}, {3., 11.8932}}	{98.783, 52.62}	inf	25.	72.2607	{18.1274, 107.571}	203.29	0.358998	newly	9.	56.25	{10., 81.}	530.333	0.603516	Reff	1.19468	0.63274	{0.17291, 1.04306}	60.3698	ar	465.	1838.74	{1633., 2102.}	295.428
5	R0	{1.42222, 6.15886}	{2.63489, 6.53401}	{{1.83414, 2.59511}, {3., 11.0075}}	{87.2648, 39.6682}	inf	35.	45.7914	{3.86363, 66.5779}	131.891	0.471124	newly	37.	58.14	{2., 87.}	168.378	0.601725	Reff	0.924443	0.50659	{0.0784525, 0.968558}	56.6078	ar	490.	1779.1	{1619., 2026.}	263.082
6	R0	{1.24363, 9.95596}	{2.47391, 6.14919}	{{2.13475, 0.505461}, {3., 9.61776}}	{104.493, 43.8195}	inf	46.	70.0745	{17.7712, 110.359}	96.4793	0.284289	newly	37.	109.26	{12., 192.}	240.27	0.38711	Reff	1.06828	0.625996	{0.104065, 2.13261}	67.6647	ar	411.	1845.02	{1648., 2507.}	348.91
7	R0	{1.36478, 5.96056}	{2.43595, 7.5877}	{{1.68866, 3.43452}, {3., 15.2638}}	{83.3413, 62.0701}	inf	43.	90.1194	{8.17757, 214.636}	150.295	0.323713	newly	48.	89.32	{9., 155.}	161.458	0.431111	Reff	1.03723	0.501365	{0.135789, 0.748294}	63.2803	ar	484.	1919.75	{1677., 2538.}	296.643
8	R0	{1.36335, 17.6429}	{2.72266, 4.74864}	{{2.46641, 1.62039}, {3., 9.73567}}	{99.7189, 73.0847}	inf	38.	37.0169	{3.25769, 33.8594}	95.0853	0.446114	newly	14.	48.24	{4., 72.}	275.857	0.379999	Reff	1.1765	0.470311	{0.152485, 0.767637}	64.3825	ar	484.	1645.13	{1524., 1724.}	239.903
9	R0	{1.26575, 5.28707}	{2.6668, 6.02545}	{{2.31298, 1.217}, {3., 10.2476}}	{113.668, 60.0049}	inf	37.	72.8552	{3.7781, 139.259}	147.915	0.394798	newly	42.	86.17	{4., 278.}	177.976	0.552505	Reff	0.882228	0.473912	{0.0980105, 0.612772}	60.8865	ar	472.	1757.94	{1553., 2430.}	272.445
10	R0	{1.42942, 9.49905}	{2.51579, 6.86429}	{{0.0512999, 0.0211061}, {3., 9.10807}}	{100.608, 27.7371}	inf	37.	59.8833	{7.60828, 75.0631}	128.346	0.397115	newly	27.	50.06	{5., 57.}	153.63	0.434072	Reff	0.86337	0.393535	{0.0195479, 0.569181}	60.2648	ar	468.	1685.93	{1550., 1741.}	260.241
11	R0	{1.46827, 15.0983}	{2.64027, 4.62649}	{{2.09384, 2.06288}, {3., 7.908}}	{79.8217, 69.3575}	inf	35.	36.6564	{2.65543, 36.2923}	118.019	0.529692	newly	18.	48.2	{2., 75.}	230.333	0.414861	Reff	1.25831	0.551988	{0.078754, 1.23645}	59.6718	ar	495.	1651.51	{1508., 1850.}	233.638
12	R0	{1.25751, 13.279}	{2.07397, 5.64395}	{{0.0566541, 0.185738}, {3., 10.6888}}	{116.731, 57.4972}	inf	31.	55.7045	{10.0314, 62.693}	116.265	0.266848	newly	18.	53.49	{9., 46.}	221.833	0.332206	Reff	1.09906	0.321452	{0.00819786, 0.525984}	76.007	ar	366.	1695.65	{1572., 1764.}	363.292
13	R0	{1.3425, 9.16361}	{2.62328, 5.97061}	{{1.20937, 3.31434}, {3., 9.27321}}	{99.1591, 38.2977}	inf	30.	54.5624	{11.9391, 63.6382}	99.9259	0.239258	newly	15.	53.61	{16., 96.}	267.133	0.436789	Reff	0.839063	0.485844	{0.10587, 0.749896}	55.2708	ar	442.	1700.1	{1585., 1921.}	284.638
14	R0	{1.18936, 6.20583}	{2.69425, 6.84428}	{{1.86188, 3.40714}, {3., 12.4824}}	{126.53, 49.1849}	inf	36.	45.4807	{6.39212, 56.4036}	122.686	0.429761	newly	30.	53.96	{4., 47.}	174.533	0.478365	Reff	0.848014	0.528859	{0.0844314, 1.1325}	61.2819	ar	382.	1760.51	{1610., 2023.}	360.866
15	R0	{1.27137, 9.78868}	{2.56456, 6.03809}	{{1.84406, 2.7622}, {3., 10.8671}}	{107.208, 42.6311}	inf	42.	45.6286	{5.42393, 46.452}	115.611	0.486635	newly	20.	66.82	{4., 60.}	293.	0.415401	Reff	0.943357	0.530998	{0.170368, 1.00791}	53.2493	ar	496.	1761.88	{1616., 1860.}	255.218
16	R0	{1.23102, 13.6452}	{2.82008, 8.06394}	{{2.4392, 5.76755}, {3., 11.1792}}	{129.085, 42.5033}	inf	28.	33.7556	{13.4114, 54.771}	80.6781	0.246688	newly	7.	29.99	{6., 39.}	339.571	0.422271	Reff	0.984816	0.507852	{0.141519, 0.794937}	57.5589	ar	445.	1690.11	{1581., 1916.}	279.8
17	R0	{1.27213, 13.7452}	{2.63856, 8.32412}	{{2.24903, 1.06611}, {3., 16.1872}}	{112.767, 47.7441}	inf	25.	41.8888	{4.66665, 53.0098}	110.217	0.308962	newly	15.	70.35	{3., 37.}	408.6	0.412921	Reff	1.11566	0.530984	{0.0802932, 1.0857}	62.6058	ar	367.	1792.3	{1643., 2057.}	388.365
18	R0	{1.25318, 11.2151}	{2.72195, 4.12898}	{{2.15049, 2.53606}, {3., 5.85102}}	{117.203, 64.4282}	inf	34.	36.5529	{2.57611, 28.9634}	124.998	0.588164	newly	18.	54.5	{3., 52.}	266.222	0.412693	Reff	0.988759	0.462113	{0.101586, 0.956239}	66.7123	ar	416.	1646.41	{1518., 1804.}	295.772
19	R0	{1.46006, 8.11496}	{2.59654, 6.98391}	{{2.23864, 0.90909}, {3., 14.4482}}	{85.7225, 58.4142}	inf	40.	33.5238	{4.02749, 36.7099}	75.9079	0.450518	newly	35.	48.72	{3., 44.}	135.657	0.527228	Reff	0.995761	0.526016	{0.213699, 0.909107}	56.3325	ar	496.	1776.83	{1651., 1798.}	258.232
20	R0	{1.14273, 6.71261}	{2.83249, 7.31695}	{{2.45479, 0.51906}, {3., 10.2455}}	{147.871, 41.1123}	inf	37.	42.2427	{6.01335, 77.1456}	90.4883	0.385838	newly	33.	43.95	{4., 100.}	122.455	0.470688	Reff	0.861618	0.491818	{0.130216, 0.791451}	56.7291	ar	362.	1754.7	{1619., 2109.}	384.724
21	R0	{1.42432, 8.23905}	{2.79599, 4.43378}	{{2.48442, 1.39572}, {3., 8.29792}}	{96.4766, 46.4496}	inf	21.	33.7244	{2.91486, 26.9267}	121.484	0.322411	newly	20.	34.	{2., 40.}	124.1	0.358914	Reff	1.02409	0.507527	{0.18082, 1.12673}	63.4786	ar	405.	1726.36	{1588., 2078.}	326.262
22	R0	{1.29386, 17.863}	{2.69678, 6.5675}	{{1.8182, 2.44169}, {3., 15.4993}}	{108.429, 63.7638}	inf	30.	42.3637	{4.04338, 49.2047}	122.404	0.424385	newly	13.	58.47	{3., 72.}	391.462	0.43854	Reff	1.13601	0.587439	{0.0908126, 1.46276}	62.5921	ar	488.	1757.3	{1591., 2222.}	260.102
23	R0	{1.39274, 13.0167}	{2.19852, 7.16721}	{{0.0984638, 3.54383}, {3., 13.9729}}	{104.299, 49.3033}	inf	50.	80.4312	{10.6927, 59.356}	131.898	0.363194	newly	23.	66.53	{9., 100.}	230.13	0.372365	Reff	0.997202	0.346804	{0.0162657, 0.533566}	66.0097	ar	475.	1693.15	{1531., 1979.}	256.453
24	R0	{1.32178, 15.8991}	{2.63673, 3.38912}	{{1.98221, 0.684746}, {3., 8.29272}}	{99.4833, 78.9731}	inf	70.	30.9888	{2.45998, 44.6393}	79.6084	0.739876	newly	28.	47.17	{3., 69.}	134.75	0.476369	Reff	1.00059	0.529979	{0.146046, 1.0182}	65.5522	ar	471.	1686.59	{1547., 1790.}	258.087
25	R0	{1.37568, 17.8427}	{2.63997, 10.8171}	{{2.4386, 6.53175}, {3., 15.0929}}	{99.7262, 39.3751}	inf	28.	62.458	{11.3722, 56.8078}	147.835	0.237513	newly	9.	35.92	{5., 34.}	314.667	0.412144	Reff	1.11843	0.474208	{0.111523, 0.750259}	70.3296	ar	398.	1702.08	{1511., 2127.}	327.658
26	R0	{1.20993, 6.4656}	{2.62844, 7.25744}	{{2.00048, 2.05331}, {3., 17.1267}}	{117.239, 74.2328}	inf	26.	49.9555	{5.00844, 80.2399}	158.855	0.44061	newly	24.	66.97	{2., 120.}	259.542	0.547387	Reff	0.908657	0.563587	{0.175571, 1.41933}	65.4017	ar	345.	1741.36	{1554., 2393.}	404.742
27	R0	{1.20548, 13.8003}	{2.52981, 5.88411}	{{2.08604, 3.0866}, {3., 15.6848}}	{115.176, 64.2429}	inf	33.	27.9802	{2.72437, 51.1641}	80.1132	0.482792	newly	20.	40.71	{3., 55.}	169.45	0.431065	Reff	0.947507	0.511128	{0.0554426, 1.15529}	65.2848	ar	350.	1639.17	{1508., 1927.}	368.334
28	R0	{1.3176, 14.6594}	{2.6587, 5.33931}	{{1.79423, 1.92086}, {3., 11.1364}}	{103.74, 67.9127}	inf	28.	36.5297	{2.77598, 42.9971}	110.158	0.435084	newly	16.	48.84	{3., 65.}	255.	0.450406	Reff	1.18979	0.536187	{0.0899698, 1.35015}	62.3446	ar	362.	1747.37	{1620., 2037.}	382.699
29	R0	{1.28202, 6.23047}	{2.63486, 3.18268}	{{1.98746, 0.875473}, {3., 5.51098}}	{105.524, 59.8115}	inf	21.	26.7483	{2.75891, 31.0171}	80.4353	0.34062	newly	22.	53.59	{3., 76.}	193.773	0.436743	Reff	0.937157	0.641819	{0.144452, 1.98396}	67.5475	ar	391.	1703.47	{1546., 2200.}	335.67
30	R0	{1.28177, 8.83252}	{2.66634, 6.24066}	{{2.27049, 0.41276}, {3., 15.8932}}	{113.22, 59.2793}	inf	41.	42.035	{4.99858, 43.9359}	93.1859	0.446042	newly	27.	64.08	{3., 43.}	204.222	0.387997	Reff	0.9562	0.49995	{0.147415, 0.757003}	61.8438	ar	427.	1751.98	{1628., 1752.}	310.3
31	R0	{1.35886, 5.97708}	{2.31947, 6.36907}	{{0.77364, 3.38318}, {3., 11.0232}}	{92.6965, 33.7284}	inf	33.	104.682	{11.6149, 196.523}	245.082	0.428154	newly	34.	99.12	{6., 179.}	228.294	0.439227	Reff	0.841134	0.523622	{0.0767885, 0.923825}	50.7851	ar	491.	1874.85	{1627., 2426.}	281.843
32	R0	{1.27601, 17.9804}	{2.62758, 3.80729}	{{1.97465, 1.8945}, {3., 7.83983}}	{105.922, 78.8253}	inf	45.	39.2664	{3.26701, 91.9256}	82.079	0.489586	newly	24.	79.12	{4., 250.}	272.083	0.459882	Reff	1.09737	0.604352	{0.149892, 1.96756}	65.8805	ar	445.	1749.5	{1535., 2386.}	293.148
33	R0	{1.3368, 11.3235}	{2.8079, 7.66399}	{{2.44238, 3.59666}, {3., 12.3052}}	{110.046, 42.09}	inf	59.	40.2517	{10.3553, 61.9613}	79.5507	0.480693	newly	32.	54.51	{6., 135.}	160.594	0.476469	Reff	0.974527	0.521269	{0.124297, 0.75799}	62.972	ar	417.	1756.01	{1606., 2243.}	321.106
34	R0	{1.21021, 11.3635}	{2.70919, 7.56227}	{{2.04008, 3.17568}, {3., 10.2423}}	{123.861, 36.3409}	inf	17.	36.7184	{5.42172, 90.7115}	152.92	0.354262	newly	9.	39.02	{3., 118.}	366.222	0.496846	Reff	1.00689	0.57328	{0.0905342, 1.64151}	73.1672	ar	327.	1746.1	{1565., 2386.}	433.976
35	R0	{1.53382, 10.8866}	{2.29101, 4.92016}	{{0.88348, 0.0990901}, {3., 9.10925}}	{74.0435, 57.2373}	inf	51.	65.4779	{5.61737, 105.256}	118.348	0.454978	newly	30.	87.31	{5., 249.}	250.767	0.466322	Reff	1.1565	0.461811	{0.114841, 0.753534}	65.4034	ar	476.	1803.01	{1612., 2153.}	278.784
36	R0	{1.20423, 6.83844}	{2.65118, 9.46422}	{{2.22494, 3.82931}, {3., 15.9206}}	{124.15, 51.3024}	inf	51.	57.3236	{12.3111, 57.528}	94.4777	0.361981	newly	46.	44.06	{6., 59.}	106.217	0.530702	Reff	0.814059	0.477497	{0.155797, 0.700536}	53.2947	ar	416.	1750.81	{1624., 1854.}	320.868
37	R0	{1.43629, 10.1548}	{2.62431, 7.01861}	{{2.01346, 3.94895}, {3., 10.0627}}	{82.7142, 31.0493}	inf	42.	62.6395	{4.92478, 120.399}	108.937	0.360295	newly	33.	66.07	{4., 139.}	166.273	0.455995	Reff	1.10594	0.602137	{0.137337, 1.99663}	71.2481	ar	367.	1780.4	{1571., 2426.}	385.123
38	R0	{1.37504, 8.93428}	{2.67401, 6.50871}	{{2.27183, 0.718255}, {3., 16.4449}}	{99.7127, 61.9237}	inf	75.	44.4746	{5.91895, 63.2284}	78.7528	0.557223	newly	60.	82.19	{4., 70.}	149.05	0.587581	Reff	0.897901	0.522255	{0.131766, 0.770711}	60.6108	ar	483.	1767.03	{1615., 1893.}	265.845
39	R0	{1.51637, 9.36742}	{2.2447, 8.69714}	{{0.913447, 1.25725}, {3., 18.2049}}	{73.0173, 61.7057}	inf	30.	46.805	{6.84862, 77.3636}	119.5	0.360812	newly	16.	45.15	{4., 86.}	236.438	0.465576	Reff	1.019	0.456908	{0.141482, 0.785941}	62.7686	ar	441.	1767.52	{1607., 2061.}	300.798
40	R0	{1.23658, 9.82869}	{2.30498, 5.22519}	{{0.638464, 1.12953}, {3., 14.1753}}	{110.345, 63.8787}	inf	71.	51.5426	{6.64959, 127.904}	82.2218	0.525614	newly	51.	68.86	{5., 126.}	118.392	0.468097	Reff	0.868079	0.438071	{0.0979283, 0.670569}	57.7429	ar	414.	1797.9	{1660., 2052.}	334.275
41	R0	{1.24247, 5.4242}	{2.5101, 6.64899}	{{1.63579, 2.98731}, {3., 12.0957}}	{103.647, 56.2433}	inf	30.	67.5426	{6.21933, 151.269}	169.451	0.390054	newly	38.	69.21	{6., 96.}	155.974	0.476563	Reff	0.956702	0.427112	{0.06429, 0.674815}	62.721	ar	345.	1806.73	{1640., 2026.}	423.69
42	R0	{1.28208, 4.40223}	{2.24707, 5.12931}	{{0.607309, 1.63945}, {3., 10.4199}}	{102.473, 62.9508}	inf	25.	46.7966	{6.40082, 41.6571}	133.891	0.367308	newly	44.	53.5	{3., 63.}	110.045	0.507527	Reff	0.951303	0.40714	{0.0933642, 0.652534}	64.1865	ar	339.	1779.65	{1642., 1937.}	424.971
43	R0	{1.56773, 6.50474}	{2.56083, 5.56021}	{{1.26248, 1.0252}, {3., 9.83915}}	{72.1063, 50.2168}	inf	43.	63.2933	{8.14553, 112.371}	87.5607	0.290941	newly	49.	64.48	{7., 114.}	98.8571	0.380918	Reff	0.981399	0.445601	{0.0732689, 0.73006}	68.0559	ar	498.	1723.61	{1593., 2202.}	248.801
44	R0	{1.10489, 6.01941}	{2.57969, 6.93041}	{{1.27894, 1.45589}, {3., 14.1362}}	{133.479, 53.953}	inf	33.	27.599	{4.5266, 26.677}	75.7572	0.415349	newly	35.	31.94	{3., 35.}	104.4	0.571244	Reff	0.79994	0.474965	{0.117632, 0.699639}	61.208	ar	346.	1669.23	{1556., 1921.}	382.436
45	R0	{1.25888, 14.1926}	{2.63057, 6.79203}	{{2.12761, 0.303311}, {3., 15.4551}}	{110.236, 56.339}	inf	65.	39.6183	{5.08087, 42.238}	67.1299	0.486223	newly	24.	58.75	{3., 77.}	197.042	0.446031	Reff	0.815754	0.543606	{0.149855, 0.962433}	58.6915	ar	471.	1666.24	{1536., 1811.}	253.766
46	R0	{1.45292, 16.616}	{2.62753, 7.50109}	{{2.24426, 0.237952}, {3., 15.8021}}	{89.1608, 55.2211}	inf	45.	41.1812	{4.60736, 53.2155}	85.9795	0.452665	newly	23.	60.86	{4., 55.}	231.565	0.424041	Reff	1.20738	0.557829	{0.12689, 1.02068}	60.7165	ar	494.	1770.72	{1624., 2062.}	258.445
47	R0	{1.22528, 16.6033}	{2.39318, 5.61503}	{{0.896038, 0.764038}, {3., 10.8372}}	{108.686, 66.449}	inf	28.	41.7684	{6.19988, 89.8966}	103.169	0.306614	newly	12.	77.87	{4., 148.}	578.25	0.490442	Reff	1.1101	0.570389	{0.123504, 1.33912}	62.3113	ar	368.	1783.96	{1627., 2207.}	384.772
48	R0	{1.1669, 6.92715}	{2.63237, 6.91716}	{{2.24419, 3.03966}, {3., 14.1118}}	{127.947, 43.0443}	inf	37.	62.9959	{8.62508, 99.8522}	117.449	0.317534	newly	38.	82.41	{9., 194.}	180.605	0.390077	Reff	0.839001	0.645038	{0.111136, 1.66076}	68.9287	ar	416.	1799.98	{1581., 2393.}	332.688
49	R0	{1.32749, 16.0887}	{2.81482, 7.05221}	{{2.42568, 3.61452}, {3., 17.9341}}	{112.041, 59.7549}	inf	27.	32.3855	{4.107, 55.0909}	79.1352	0.338853	newly	11.	38.74	{3., 52.}	288.909	0.455302	Reff	1.17085	0.446038	{0.136309, 0.577223}	70.9188	ar	353.	1731.9	{1582., 2071.}	390.623
50	R0	{1.29636, 15.2609}	{2.77576, 4.29183}	{{2.46036, 0.428389}, {3., 12.2369}}	{115.597, 71.8769}	inf	42.	32.879	{4.89436, 44.5076}	80.0176	0.45272	newly	19.	56.77	{6., 76.}	240.263	0.369728	Reff	0.917823	0.57266	{0.184377, 1.02877}	57.9679	ar	470.	1655.57	{1520., 1784.}	252.249
